# Supplementary material for: The protective effect and immunomodulatory ability of orally administrated Lacticaseibacillus rhamnosus GG against Mycoplasma pneumoniae infection in BALB/c mice
Source: PLoS One. 2024 Oct 25;19(10):e0312318. doi: 10.1371/journal.pone.0312318 (PMC11508164; doi:10.1371/journal.pone.0312318)
Supplement: Attachment — Submitted filename: Response to Reviewers.docx [file pone.0312318.s001.docx]

Manuscript ID: PONE-D-24-11070 ---Revised for PLOS ONE.

Entitled " *The protective effect and immunomodulatory ability of orally administrated* *Lacticaseibacillus rhamnosus GG against Mycoplasma pneumoniae infection in BALB/c mice”* by Long H *et al.*

We would like to thank the reviewers and the editor for the good comments on our manuscript. Following the comments, we have revised our manuscript and carefully checked and edited English grammar, spelling, and sentence structure by a professional language editor. Moreover, we supplement the experiment of “The investigation of intestinal supplementation with *Five strains of lactobacillus* on *Mycoplasma pneumoniae* infection in mouse models”. Hopefully, you will find our revised manuscript suitable for publication in PLOS ONE. Our point-to-point responses to the reviewers' comments are listed as follows.

**Reviewer 1 comments：**

1. According to the International Scientific Association for Probiotics and Prebiotics, the name "Lactobacillus rhamnosus" should be changed to "Lacticaseibacillus rhamnosus."

Response: Thank you for your correction. We have replaced "*Lactobacillus rhamnosus* GG" with "*Lacticaseibacillus rhamnosus* GG".

2. The animals were maintained under SPF conditions. Were they born under the same conditions? Did you use SPF mice?

Response: The BALB/c mice used in this experiment were purchased from Gempharmatech Co., Ltd, which has an SPF-level experimental animal production license and an experimental animal usage license. Mice are packed in special autoclaved cardboard with transparent viewing windows and filter membranes. Then mice were housed in the SPF Animal Laboratory at the University of South China. So, the mice used in our experiment are SPF mice.

3. For the anti-*M. pneumoniae* antibodies evaluation (lines 160-161), please include the dilution employed for serum and BAL (probably BAL was used undiluted, but serum must have been diluted). Moreover, signals look quite low, especially for serum IgM (of course, it depends on the dilution factor). Can you re-evaluate serum antibodies, including some samples of normal, uninfected mice? Including a line in the graph showing the "background" signal of the ELISA would be helpful in understanding if there are no differences in specific antibodies produced or if there are no specific antibodies produced.

We express our sincere appreciation for your valuable advice. In our endeavor to detect anti-M. pneumoniae antibodies, we employed undiluted BAL fluid and a 1:2 dilution for the serum. Given the low immunogenicity of *M. pneumoniae*, the resultant antibody detection signals are correspondingly low ^[1,2]^. In the results section, we included the levels of serum IgM and sIgA in uninfected mice, as well as the levels of macrophages and neutrophils in BAL fluid and lung tissue to illustrate the "background" signal.

Reference:

[1] Chu HW, et al. Repeated respiratory *Mycoplasma pneumoniae* infections in mice: effect of host genetic background. Microbes Infect, 2006 Jun;8(7):1764-72. doi: 10.1016/j.micinf.2006.02.014.

 [2] Wubb el L, et al. Mycoplasma pneumoniae pneumonia in a mouse model. J Infect Dis

. 1998 Nov;178(5):1526-9. doi: 10.1086/314439.

4. I assume that comparisons in brackets (t=xxxx, p) The authors claim that specific molecules might be involved in the effects of probiotics since even the heat-killed bacteria present those effects. They suggest that peptidoglycan and exopolysaccharide may explain the effects. However, exopolysaccharides are molecules commonly secreted to the medium, and they cannot be presented at high concentration in heat-killed bacteria. I suggest exploring the potential role of **lipoteichoic acid instead of exopolysaccharide** (Nutrients Volume 14, Issue 3, 2022 Article number 723: is a research of my laboratory, the reference is not to cite in this manuscript, but just to support my comment).

Response: We sincerely appreciate the valuable comments. We re-analyzed the causes of the heat-killed LGG present anti-*M. pneumoniae* effects, and replaced exopolysaccharide to lipoteichoic acid, also provide the reference. Moreover, lipoteichoic acid could be a valuable line of thought for our next experiment.

5.Line 91 "...(MRS) medium and LACATE dehydrogenase (LDH)..."

Response: We have replaced "Lacate" with " LACATE ".

**Reviewer 2 comments：**

1. The main concern is the lack of a non-challenge control group in the experiment. The study only includes groups challenged with M. pneumoniae but does not have a non-challenge control. The absence of data from normal mice makes interpreting the results challenging.

Response: Thank you for pointing it out. We had a non-challenge control group in the experiment. We are sorry the results of the non-challenge control group were not arranged. We supplemented the result of the non-challenge control group in the revised manuscript.

2. Another concern is the inconsistency in the timing of data collection. Data presented in Figs 1-4 are at 3 dpi, while data in Figs 5-7 are at 7 dpi. This inconsistency makes it difficult to correlate the factors affecting the outcomes. It would be better to include testing for Figs 1-4 at 7 days and for Figs 5-7 at 3 days.

Response: At 3 dpi, this specific day point was chosen because it coincided with the peak of lung tissue inflammation and the highest organism loads in the infected mouse lungs. At 7 dpi, to examine the humoral immunity of *M. pneumoniae*-infected mice with or without LGG supplementation and to determine whether administering LGG affects T-cell-mediated immunity during *M. pneumoniae* infection, adaptive immunity detection typically occurred 7 days after M. pneumoniae infection, or even an extended duration was needed.

3. L50 Lacticaseibacillus should be Lactobacillus.

Response: According to the International Scientific Association for Probiotics and Prebiotics, the name "*Lactobacillus casei*" was changed to “*Lacticaseibacillus casei*”. *Lacticaseibacillus casei* CNRZ1874 was abbreviated as *L. casei* CNRZ1874 in the revised manuscript.

4. L52. Please provide the reference related the safety of L. casei.

Response: We have supplemented some information on the safety of *L. casei* and provided the related reference. *L. casei* is a common probiotic genus on the qualified presumption of the safety (QPS) list in Europe and China ^[1]^. However, it is well known, that the probiotic effects are strain-specific, further assessment of the safety of using *L. casei* CNRZ1874 is necessary.

Reference:

[1] EFSA BIOHAZ Panel, Koutsoumanis, K., Allende, A., Alvarez- Ordonez, A., Bolton, D., Bover- Cid, S. et al. (2022) Updated list of QPS- recommended biological agents for safety risk assessments carried out by EFSA.

5. L104. How was the efficacy of the heat inactivation of LGG verified?

Response: After the LGG was assessed at 70°C for 1 h using a temperature-controlled water bath, the organism was cultured in MRS solid culture for 48 hours, and no colony was observed. This is according to the results of the literature (doi: 10.1016/j.ijfoodmicro.2013.04.022), which showed that after 20 min heat treatment at 60 °C, colony-forming units of LGG were significantly decreased. The temperatures 70 °C and 80 °C were already lethal for strains ^[1]^.

Reference:

[1] du Toit E, Vesterlund S, Gueimonde M, Salminen S. Assessment of the effect of stress-tolerance acquisition on some basic characteristics of specific probiotics. Int J Food Microbiol 2013, 165(1):51-56. doi: 10.1016/j.ijfoodmicro.2013.04.022.

6. L126. The score system should be detailed.

Response: Thanks for the good comment. We have detailed described the scoring system.

7. L177. Is there any live LGG recovered or LGG gene detected in BAL from any of the three treatment groups?

Response: Thanks for the good suggestion. We did not detect BAL LGG from any of the three treatment groups, this is the deficiency of our experiment. According to literature reports, the gut epithelial barrier separates the internal intestinal milieu from the luminal environment, thereby ascertaining the permeability of nutrients and other molecules as well as exerting a protective role by arresting the entry of microbes and toxic compounds ^[1]^. Studies have reported that commensal Lactobacilli spp. maintain gut barrier integrity which depends upon the multi-protein complexes such as tight junctions, gap junctions, adherents, and desmosomes ^[2]^. Additionally, findings manifested that it was by expanding mesenteric CD103+DCs and accumulating mucosal Tregs that LGG treatment orally contributes to protecting against OVA-induced allergic airway inflammation ^[3]^. While the appropriate tests are still needed to ascertain whether LGG can be identified in BAL.

Reference:

[1] Rastogi S, Singh A. Gut microbiome and human health: Exploring how the probiotic genus Lactobacillus modulate immune responses. Front Pharmacol. 2022 Oct 24;13:1042189. doi: 10.3389/fphar.2022.1042189.

[2] Kocot AM, Jarocka-Cyrta E, Drabińska N. Overview of the Importance of Biotics in Gut Barrier Integrity. Int J Mol Sci. 2022 Mar 7;23(5):2896. doi: 10.3390/ijms23052896.

[3] Zhang J, Ma JY, Li QH, Su H, Sun X. Lactobacillus rhamnosus GG induced protective effect on allergic airway inflammation is associated with gut microbiota. Cell Immunol. 2018 Oct;332:77-84. doi: 10.1016/j.cellimm.2018.08.002.

8. Figue 5 How about the IgG levels against Mp? This data is collected at 7 dpi and might not be relevant to the positive effects observed at 3 dpi.

Response: IgM is typically the first antibody to be produced after *M. pneumoniae* infection. In the *M. pneumoniae*-infected BALB/c mice model, IgM peaked on day 7 after a primary infection. IgG is the major antibody class seen late after the first infection, the reaction of mycoplasma with mycoplasma-specific IgG is necessary for phagocytosis of the organism by macrophages (opsonization). Generally, a noticeable IgG response was observed at day 14 post *M. pneumoniae* infection in BALB/c mice^[1,2]^, so at 7 dpi, we did not detect the IgG levels. Thanks for the good comment, in our future studies, we will further refine our experiment, and detect the most important specific IgG antibodies against mycoplasma infection on day 14 after the pathogen infection.

Reference:

[1] Chu HW, et al. Repeated respiratory *Mycoplasma pneumoniae* infections in mice: effect of host genetic background. Microbes Infect, 2006 Jun;8(7):1764-72. doi: 10.1016/j.micinf.2006.02.014.

 [2] Wubb el L, et al. Mycoplasma pneumoniae pneumonia in a mouse model. J Infect Dis

. 1998 Nov;178(5):1526-9. doi: 10.1086/314439.

9. L312. It might need to test at a longer time point instead of 7 days.

Response: Thank you for your good suggestion. In the *M. pneumoniae*-infected BALB/c mice model, IgM peaks on day 7 after a primary infection. But at this time point, IgG, the most important antibody against mycoplasma infection, is almost undetectable on 7 dpi. A longer time point instead of 7 dpi is needed. In future experiments, we will test the antibodies on day 14 dpi or a longer point.
